# Prevalence of *Mycobacterium lentiflavum* in cystic fibrosis patients, France

**DOI:** 10.1186/s12890-015-0123-y

**Published:** 2015-10-26

**Authors:** Michael Phelippeau, Jean-Christophe Dubus, Martine Reynaud-Gaubert, Carine Gomez, Nathalie Stremler le Bel, Marielle Bedotto, Elsa Prudent, Michel Drancourt

**Affiliations:** Aix Marseille Université, URMITE, UMR CNRS 7278, IRD 198, INSERM 1095. Faculté de Médecine, Marseille, 13005 France; Centre de Ressource et de Compétences de la Mucoviscidose (CRCM) pédiatrique CHU Hôpital la Timone, Marseille, France; Centre de Ressource et de Compétences de la Mucoviscidose (CRCM) adulte; équipe de Transplantation pulmonaire, CHU Hôpital Nord, URMITE - CNRS-UMR 6236 Aix-Marseille Université, Marseille, France; Unité de recherche sur les maladies infectieuses et tropicales émergentes, Faculté de Médecine, 27 Bd jean Moulin, 13385 Marseille, cedex 5 France

**Keywords:** *Mycobacterium lentiflavum*, Cystic fibrosis, *rpoB*, *smpB*

## Abstract

**Background:**

*Mycobacterium lentiflavum* is rarely isolated in respiratory tract samples from cystic fibrosis patients. We herein describe an unusually high prevalence of *M. lentiflavum* in such patients.

**Methods:**

*M. lentiflavum,* isolated from the respiratory tract of cystic fibrosis patients, was identified using both *rpoB* partial sequencing and detected directly in the sputum by using real-time PCR targeting the *smpB* gene.

**Results:**

*M. lentiflavum* emerged as the third most prevalent nontuberculous mycobacterial species isolated in cystic fibrosis patients in Marseille, France. Six such patients were all male, and two of them may have fulfilled the American Thoracic Society clinical and microbiological criteria for *M. lentiflavum* potential lung infection.

**Conclusions:**

*M. lentiflavum* was the third most common mycobacteria isolated in cystic fibrosis patients, particularly in six male patients. *M. lentiflavum* outbreaks are emerging particularly in cystic fibrosis patients.

**Electronic supplementary material:**

The online version of this article (doi:10.1186/s12890-015-0123-y) contains supplementary material, which is available to authorized users.

## Background

*Mycobacterium lentiflavum* is a fastidious nontuberculous mycobacterium (NTM) isolated from the respiratory tract, urine, lymph nodes and vertebral-bone specimens [1–4]. *M. lentiflavum* has seldom been reported in cystic fibrosis patients, at a much lower prevalence than *Mycobacterium abscessus* and *Mycobacterium avium* [5–8]. Moreover, its clinical significance is debated because *M. lentiflavum* is an environmental organism [9].

As we observed an unusual prevalence of *M. lentiflavum* isolates in clinical samples taken from patients suffering from respiratory diseases, the objective of this clinical study was to describe the potential opportunistic role of *M. lentiflavum* in cystic fibrosis.

## Methods

### Detection and isolation of *M. lentiflavum*

Respiratory tract specimens were prospectively collected and analyzed in the Reference Laboratory for Mycobacteria of the Institut Hospitalo-Universitaire Méditerranée Infection in Marseille, France. After decontamination using 4 % NaOH-N-acetyl-L-cysteine according to the manufacturer’s recommendations (MycoPrep, Becton Dickinson, Le Pont-de-Claix, France), each specimen was centrifuged and the pellet was microscopically examined after Ziehl-Neelsen staining. A 500-μL aliquot was simultaneously inoculated into a mycobacterial growth indicator tube (MGIT, Becton Dickinson, Le Pont-de-Claix, France) and onto a Coletsos slant (bioMérieux, La-Balme-les-Grottes, France) incubated at 37 °C in a 5 % CO_2_ atmosphere. After Ziehl-Neelsen staining confirmation of positive cultures, the isolates were identified using partial *rpoB* sequencing [10].

Direct detection of *M. lentiflavum* in sputum samples was conducted using a specific real-time PCR assay. Briefly, two primers and a probe were designed to specifically hybridize the *M. lentiflavum smpB* gene (Table 1). The specificity of this assay was checked *in silico* using the Basic Local Alignment Search Tool (BLAST) [10]. *In vitro* assessment of a collection of sixteen *Mycobacterium* species (including *M. lentiflavum*) previously identified by partial *rpoB* gene sequencing [11], yielded 100 % sensitivity and 100 % specificity for *M. lentiflavum* (Additional file 1).

**Table 1:** Probe and primer sequences and protocol for real-time PCR targeting *Mycobacterium lentiflavum*

Target gene	*smpB*
Primers	
MlentismpB MBF	CAACTTGCACATTCCCGAGT
MlentismpB MBR	CCCGATCAGTGTGTCGATCT
Probe	
MlentismpB MBR	6FAM-TCGCACTCGGAAGTTGTTGTTACATAGGC
Dilution	0.1 nmol/μL then 1/40

The extraction of DNA was performed using the EZ1 DNA tissue kit with a Qiagen EZ1 extractor Advanced XL (Qiagen, Courtaboeuf, France) according to the manufacturer’s recommendations. Real-time PCR was performed using a Biorad CFX96 thermocycler with the FAST qPCR MasterMix Plus No ROX kit (Eurogentec, Angers, France) according to the manufacturer’s recommendations: five minutes at 95 °C for activation, followed by 40 cycles of 95 °C for 10 s and 60 °C for 35 secons. Amplification products were analyzed using Biorad software

### Statistical analysis

Statistical analysis was performed using EpiInfo v3.5.4 software; *p* < 0.05 was needed for statistical significance.

### Ethics

This work was approved by the IFR48 local ethics committee at the Faculty of Medicine, under reference number 07–008. No written consent was needed for this work in accordance with the ‘LOI n° 2004–800 relative à la bioéthique’ [Law No. 2004–800 concerning bioethics] published in the *Journal Officiel de la République Française* on 6 August 2004 because no additional samples were obtained for the study.

## Results and discussion

Between January 2010 and September 2014, respiratory tract specimens (sputum, bronchoalveolar lavages and bronchial aspirates) collected from 354 cystic fibrosis patients (235 adults ≥18 years and 119 children <18 years) with a female/male ratio of 199/155 (56.2 %) were analyzed for mycobacteria (mean of 13.1 collected specimen/patient).

**Fig. 1:**
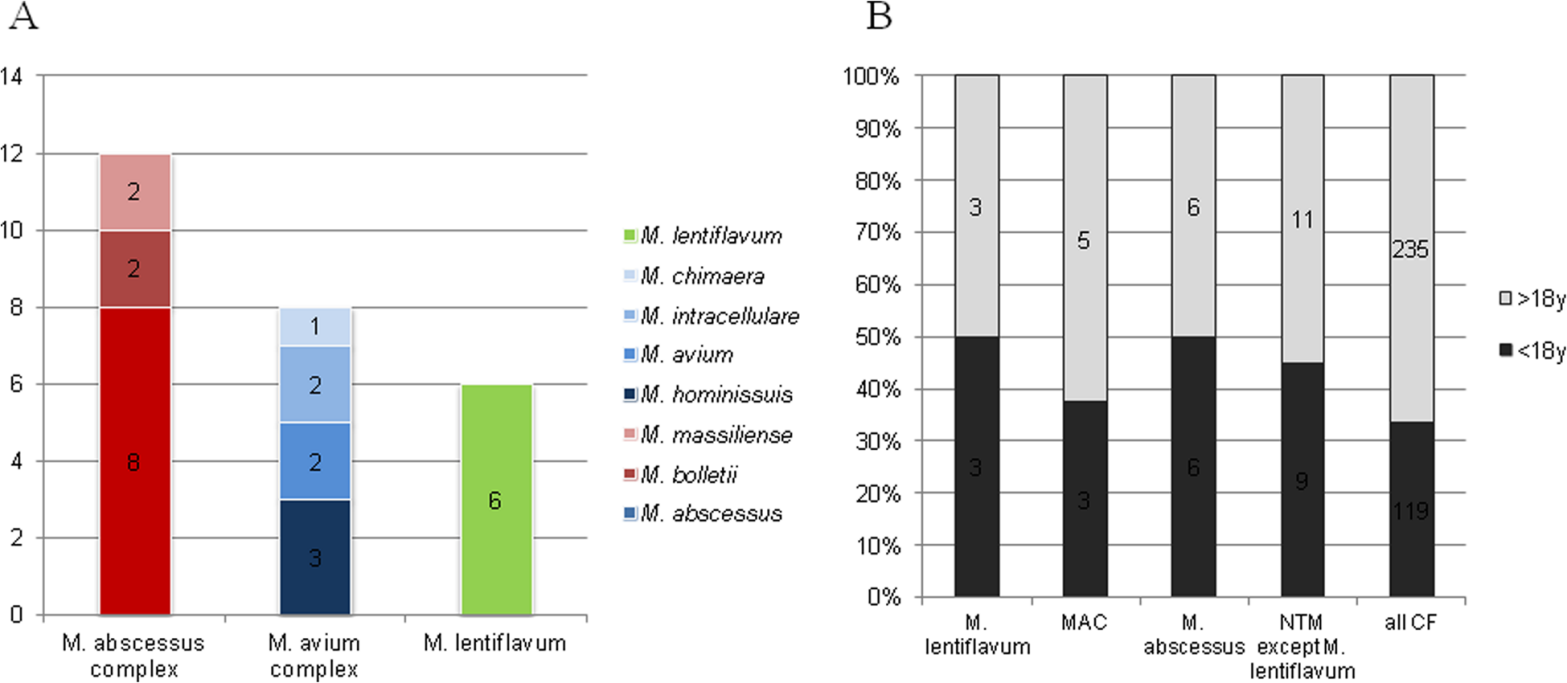
Nontuberculous mycobacteria (NTM) isolated from 25/354 cystic fibrosis (CF) patients, January 2010 to September 2014, in Marseille, France. **a** The number of the patients with at least one respiratory tract specimen that yielded NTM is shown in bars. NTM are color-coded. *M. hominissuis, Mycobacterium avium* subsp. *hominissuis; M. massiliense, Mycobacterium abscessus* subsp. *massiliense; M. bolletii*: *Mycobacterium abscessus* subsp. *bolletii*. **b** Proportion of the pediatric (<18 years) and adult (>18 years) CF patients (n_CF_ = 354) including 25 who yielded NTM including *M. lentiflavum*, *M. avium* complex and *M. abscessus* complex

In our series, 25/354 (7.1 %) cystic fibrosis patients (twelve children and thirteen adults) had at least one respiratory tract specimen that yielded NTM, including twelve (48 %) patients with *M. abscessus* complex mycobacteria, eight (32 %) patients with *M. avium* complex mycobacteria and six (24 %) patients with *M. lentiflavum* (Fig. 1); one patient had both *M. avium* and *M. lentiflavum* successively isolated during the study. A total of thirteen *M. lentiflavum* isolates were identified on the basis of 99.6 % ± 0.003 % similarity with the reference *M. lentiflavum* CIP 105465^T^ partial *rpoB* sequence [GenBank:EU109300]. The six *M. lentiflavum* patients were co-infected by *Staphylococcus aureus*, Gram-negative bacilli and fungi and were all under azithromycin long-term low-dose prophylaxis (250 mg three times a week). In two patients, *M. avium* had been previously isolated in 2004 and 2012 from respiratory tract specimens (Table 2).

**Table 2:** Clinical presentation of the six cystic fibrosis patients who yielded *Mycobacterium lentiflavum* isolates

	Mean	Standard deviation	Limits
Age, y	22.2	+/−11.4	[14.6 - 44.1]
Pedatric/Adults	3/3		
Male, %	100		
FEV, % predicted	67 %	+/−19 %	[46 – 93 %]
BMI, kg/m^2^	20	+/−1.9	[17.2 – 22.4]
Infected (met ATS criteria)^a^	2/6		
Diabetes	3/6		
Exocrine pancreatic disease	6/6		
*Pseudomonas aeruginosa*	5/6		
*Stenotrophomonas maltophilia*	4/6		
Previous NTM isolation^b^	2/6		
MS *Staphylococcus aureus*	6/6		
*Aspergillus* sp.	5/6		
Other co-infection^c^	4/6		
Azithromycin prophylaxis^d^	6/6		
Lung transplanted after isolation	1/6		

*FEV* forced expiratory volume, *ATS* American Thoracic Society, *BMI* body mass index, *NTM* nontuberculous mycobacteria, *MS* methicillin susceptible

^a^Patients who fulfilled the American Thoracic Society’s microbiological and clinical criteria for NTM pulmonary disease [12]

^b^ *Mycobacterium avium* complex

^c^ *Nocardia* sp., *Penicillium* sp., *Serratia* sp., *Achromobacter* sp., *Scedosporium* sp

^d^250mg *per os* three times a week

**Fig. 2:**
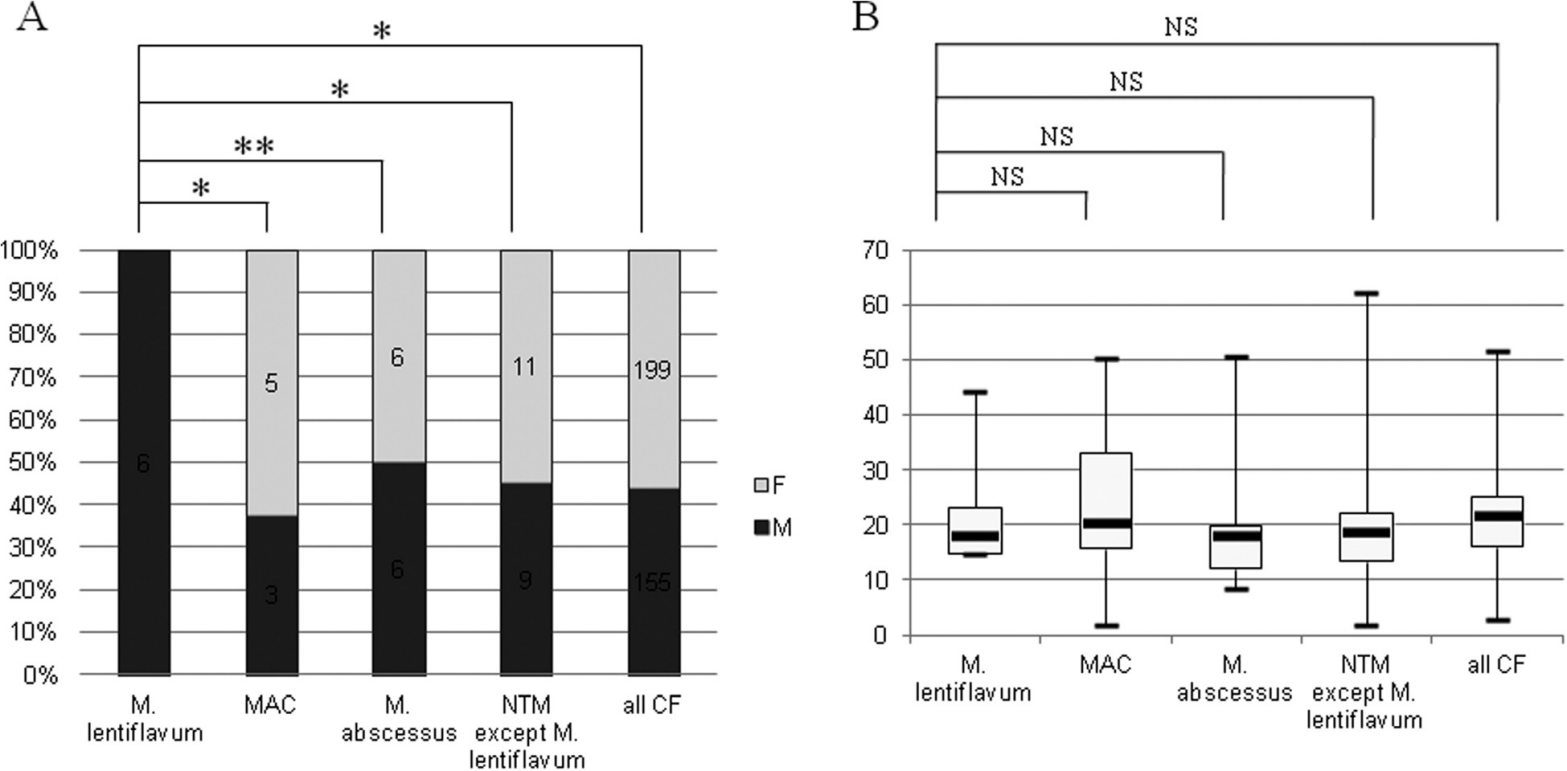
Sex-ratio (**a**) and age distribution (**b**) of the 354 cystic fibrosis (CF) patients and 25 patients who yielded nontuberculous mycobacteria (NTM) including *Mycobacterium lentiflavum* (*n* = 6), *Mycobacterium avium* complex (*n* = 8) and *Mycobacterium abscessus* complex (*n* = 12). * *p* < 0.05; ** *p* = 0.053; NS, *p* > 0.1

The female/male sex ratio of *M. lentiflavum* patients (0/6) significantly differed from that of the NTM positive cohort (199/155; 56.2 %) (*p* = 0.007, Fisher exact test) and from that of patients with another NTM (11/9; 55 %) (*p* = 0.02). It further differed from that of *M. avium* complex patients (5/3; 63 %) (*p* = 0.03) and that of *M. abscessus* complex patients (6/6; 50 %) (*p* = 0.054) (Fig. 2a). The six *M. lentiflavum* patients were aged 22.2 ± 11.4 y; the patients with another NTM were aged 22.1 ± 15.1 y; the *M. avium* complex patients were aged 26.5 ± 19.9 y; and the *M. abscessus* complex patients were aged 19.2 ± 10.9 y (no statistical significance; *p* = 0.56; ANOVA) (Fig. 2b).

Two *M. lentiflavum* patients were clinically stable, had only one positive specimen and were classified as ‘colonized’ [12]. The four other *M. lentiflavum* patients had between two and four positive sputum specimens and in two of them, *M. lentiflavum* isolation occurred contemporaneously to the decline of their lung function and thus may fulfill the American Thoracic Society’s (ATS) criteria for NTM lung infection [12] (Additional file 2: Figure S1). Thereafter, the forced expiratory volume improved during antibiotic treatment for *M. lentiflavum*. One of these two infected patients, aged 17, underwent a double lung-transplant because of poor progression of cystic fibrosis, two years after *M. lentiflavum* infection had been treated with combined rifabutin, clarithromycin and ethambutol for fourteen months and amikacin for one week. At the four-month follow-up after transplantation, microbiological surveys did not yield any further *M. lentiflavum* from four separate bronchoalveolar lavages.

In this study, we confirmed the identification of *M. lentiflavum*, a fastidious organism usually isolated over a period of three weeks, using *rpoB* partial sequencing [10] and a specific real-time PCR assay targeting the *M. lentiflavum smpB* gene. Indeed, *M. lentiflavum* shares <96 % similarity regarding the *rpoB* gene sequence with closely related species, including *Mycobacterium stomatepiae* DSM 45059, *Mycobacterium florentinum* DSM 44852, *Mycobacterium genavense* FI-06288 and *Mycobacterium triplex* ATCC 700071 [GenBank:HM022213, HM022205, HM022216 and GQ153311]. Moreover, the routinely used 16S-23S rRNA intergenic spacer sequencing [5], *hsp65* restriction fragment length polymorphism and sequencing [13], and commercial probes for *M. avium* complex [7, 8, 13] may not be sufficiently discriminative as, for example, *M. lentiflavum* shares > 99 % similarity in the 16S rRNA gene sequence with *M. simiae* [1].

*M. lentiflavum* has emerged over a five-year period as the third most prevalent NTM isolated from the respiratory tract in our cystic fibrosis cohort. Furthermore, we observed an unexpectedly higher prevalence of patients (6 out of 354; 1.7 %) showing *M. lentiflavum* isolation than other reported epidemiological surveys. Indeed, only one out of 2912 (0.03 %) French patients [5] and two out of 2970 (0.06 %) American patients [7] were reported in previous studies. In addition, a recent epidemiology survey of NTM isolated in cystic fibrosis patients in Turkey revealed nine *M. lentiflavum* isolates collected from one young male teenager out of 130 (0.8 %) cystic fibrosis patients [8]. The reason why *M. lentiflavum* has only been isolated in male cystic fibrosis patients remains unexplained. As reported by Bryant *et al.* [14], patient-to-patient contamination may be suspected although this type of cross-contamination is very rare and whole genome sequence analyses would probably be required to satisfactorily conclude a phylogenetic link and track transmission events. Moreover, the six patients described here were treated in two distinct centers (adult and pediatric) for cystic fibrosis. Environmental transmission is another hypothesis for such prevalence.

An in-lab contamination hypothesis has been proposed, and eleven non-cystic fibrosis patients yielded *M. lentiflavum* isolates (out of more than 800 patients (≈1.3 %) who yielded at least one mycobacterial isolate) during the same five-year period. This demonstrates that *M. lentiflavum* was isolated every two months on average and that the probability of in-lab cross-contamination does exist but remains low.

In order to detect *M. lentiflavum* rapidly in cystic fibrosis patients, we developed an ‘in-lab’ real-time PCR targeting the *M. lentiflavum smpB* gene. This real-time PCR proved its ability to identify all *M. lentiflavum* isolates specifically. Moreover, our preliminary results indicate that this real-time PCR may be used as a first screening step directly performed on heat-inactivated sputum specimens with good sensitivity and 100 % specificity. These results have to be compared with traditional laboratory tools (culture and AFB smears) to clarify its relevance for clinical practice [15] and have to be validated on larger series of prospectively-collected sputum specimens, including from patients who had previously yielded *M. lentiflavum* in sputum cultures.

*M. lentiflavum* had been considered to be a harmless organism. However, this interpretation was recently challenged by the publication of a few cases with disseminated *M. lentiflavum* infections [16–18]. In one case, hemophagocytic lymphohistiocytosis and disseminated *M. lentiflavum* infection in a heart-transplanted patient led to the death of this immune-compromised patient within ten days [18].

In the present study, two out of six patients fulfilled the ATS clinical and microbiological criteria for NTM lung disease [12] and had improved respiratory function while receiving specific antibiotic therapy. However, ATS criteria, while they continue to be applied to cystic fibrosis patients, are far from specific for such patients where radiographic findings, which are often associated with NTM, are commonplace irrespective of colonization. Moreover, clinical decline occurs in cystic fibrosis for a multitude of reasons. The antibiotic therapy our patient received is, moreover, active against a wide range of respiratory tract pathogens.

In our series, all patients were receiving long-term azithromycin therapy (Table 2). This use of macrolide in CF patients was shown to be a risk factor for NTM infection, especially with *M. abscessus* [19], by inhibiting intracellular killing of mycobacteria in macrophage by impairing autophagic and phagosomal degradation [20]. Such mechanisms may have played a role in the increase of *M. lentiflavum* isolation. However, as *M. lentiflavum* is usually susceptible to clarithromycin [8], the fact that patients were receiving macrolide and *M. lentiflavum* developed further supports the theory that this is either an environmental contaminant or a transient colonizer which has not been exposed to macrolide for prolonged periods.

## Conclusion

*M. lentiflavum* was the third most common NTM isolated from male cystic fibrosis patients, although few respiratory cases had been previously reported, particularly in such patients. We propose monitoring cystic fibrosis patients’ respiratory tract samples for mycobacteria detection, to achieve this goal, we propose the use and development of specific molecular tools such as *rpoB* partial sequencing (or a specific real-time PCR which needs to be fully validated against traditional laboratory tools) to monitor the presence of *M. lentiflavum* in each cystic fibrosis center and reference laboratories for mycobacteria.

## Additional files

Additional file 1: Table S1.
*Mycobacterium* species tested for the specificity/sensitivity assay of real-time PCR for *M. lentiflavum* species. (PDF 43 kb) Additional file 2: Figure S1. Clinical and microbiological data concerning two cystic fibrosis patients who yielded more than one *M. lentiflavum* isolate and who may fulfill the American Thoracic Society’s clinical and microbiological criteria for NTM lung infection. BMI: Body Mass Index. (PDF 121 kb)

## Abbreviations

AFB: Acid- Fast Bacilli
ANOVA: analysis of variance
ATS: American Thoracic Society
BLAST: Basic Local Alignment Search Tool
BMI: Body Mass Index (Supplementary fugure)
CF: cystic fibrosis
NTM: nontuberculous mycobacterium
PCR: polymerase chain reaction
