# Supplementary material for: Prevalence of Mycobacterium lentiflavum in cystic fibrosis patients, France
Source: BMC Pulm Med. 2015 Oct 26;15:131. doi: 10.1186/s12890-015-0123-y (PMC4621861; doi:10.1186/s12890-015-0123-y)
Supplement: Additional file 1: Table S1. — Mycobacterium species tested for the specificity/sensitivity assay of real-time PCR for M. lentiflavum species. (PDF 43 kb) [file 12890_2015_123_MOESM1_ESM.pdf]

Additional table. *Mycobacterium* species tested for the specificity/sensitivity assay of real-time PCR for *Mycobacterium lentiflavum* species.

Mycobacterium species	Number of isolates used	Real-time PCR positive
Mycobacterium abscessus subsp. *abscessus*	1	0
Mycobacterium abscessus subsp. *bolletii*	1	0
Mycobacterium avium	3	0
Mycobacterium avium subsp. *hominissuis*	1	0
Mycobacterium bovis Bacille Calmette–Guerin strain Tokyo	1	0
Mycobacterium chelonae	2	0
Mycobacterium chimaera	1	0
Mycobacterium europaeum	2	0
Mycobacterium fortuitum	1	0
Mycobacterium gordonae	1	0
Mycobacterium intracellulare	1	0
Mycobacterium kansasii	2	0
Mycobacterium lentiflavum (including 6 *M. lentiflavum* isolates reported in this article)	9	9
Mycobacterium porcinum	1	0
Mycobacterium simiae	2	0
Mycobacterium xenopi	1	0
TOTAL	30	9
