# Supplementary material for: Prevalence of Mycobacterium lentiflavum in cystic fibrosis patients, France
Source: BMC Pulm Med. 2015 Oct 26;15:131. doi: 10.1186/s12890-015-0123-y (PMC4621861; doi:10.1186/s12890-015-0123-y)
Supplement: Additional file 2: Figure S1. — Clinical and microbiological data concerning two cystic fibrosis patients who yielded more than one M. lentiflavum isolate and who may fulfill the American Thoracic Society’s clinical and microbiological criteria for NTM lung infection. BMI: Body Mass Index. (PDF 121 kb) [file 12890_2015_123_MOESM2_ESM.pdf]

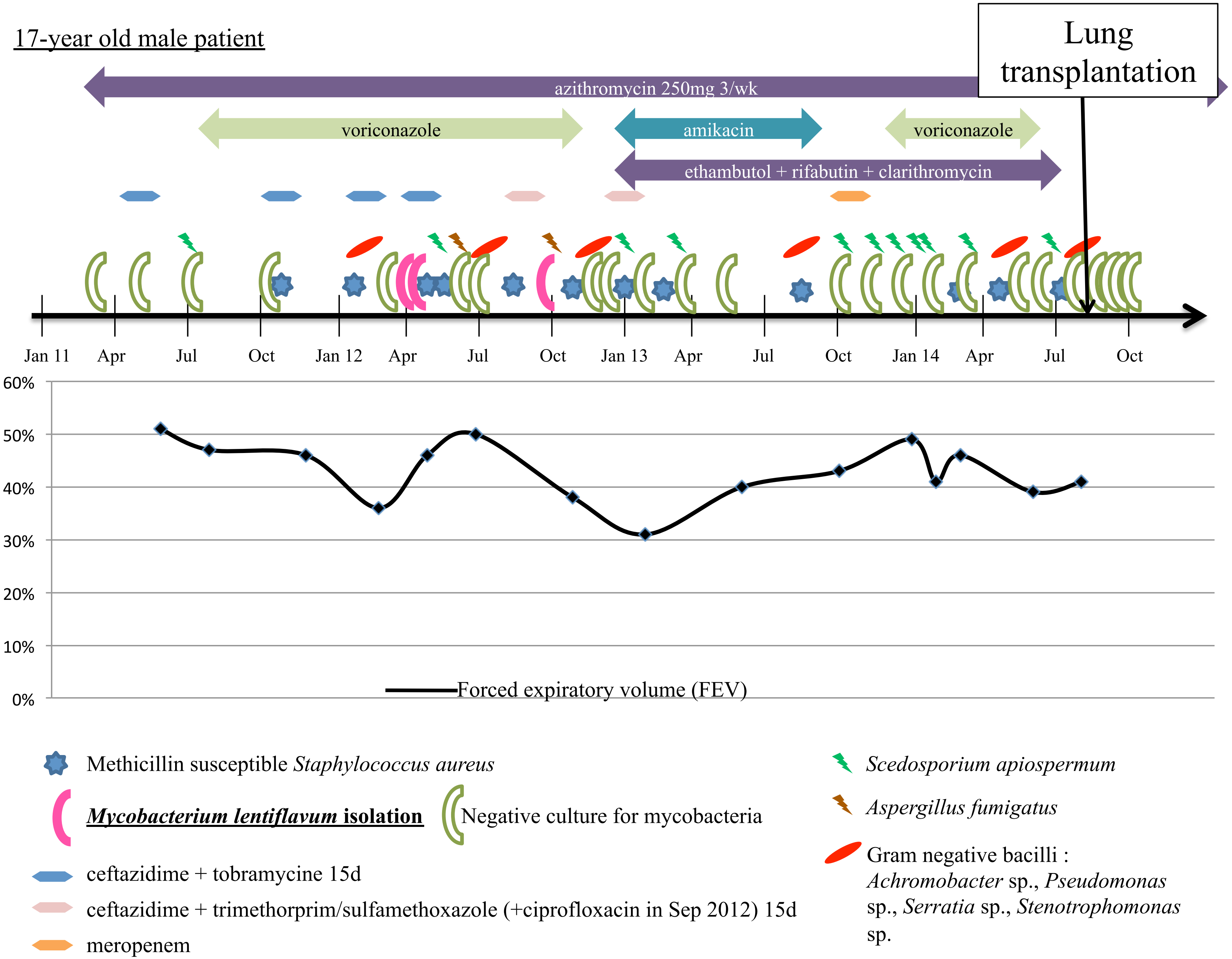
17-year old male patient
Lung transplantation
azithromycin 250mg 3/wk
voriconazole
amikacin
voriconazole
ethambutol + rifabutin + clarithromycin
Jan 11
Apr
Jul
Oct
Jan 12
Apr
Jul
Oct
Jan 13
Apr
Jul
Oct
Jan 14
Apr
Jul
Oct
60%
50%
40%
30%
20%
10%
0%
Forced expiratory volume (FEV)
Methicillin susceptible *Staphylococcus aureus*
***Mycobacterium lentiflavum* isolation**
Negative culture for mycobacteria
ceftazidime + tobramycine 15d
ceftazidime + trimethorprim/sulfamethoxazole (+ciprofloxacin in Sep 2012) 15d
meropenem
Scedosporium apiospermum
Aspergillus fumigatus
Gram negative bacilli : *Achromobacter* sp., *Pseudomonas* sp., *Serratia* sp., *Stenotrophomonas* sp.

<u>36-year old male patient</u>

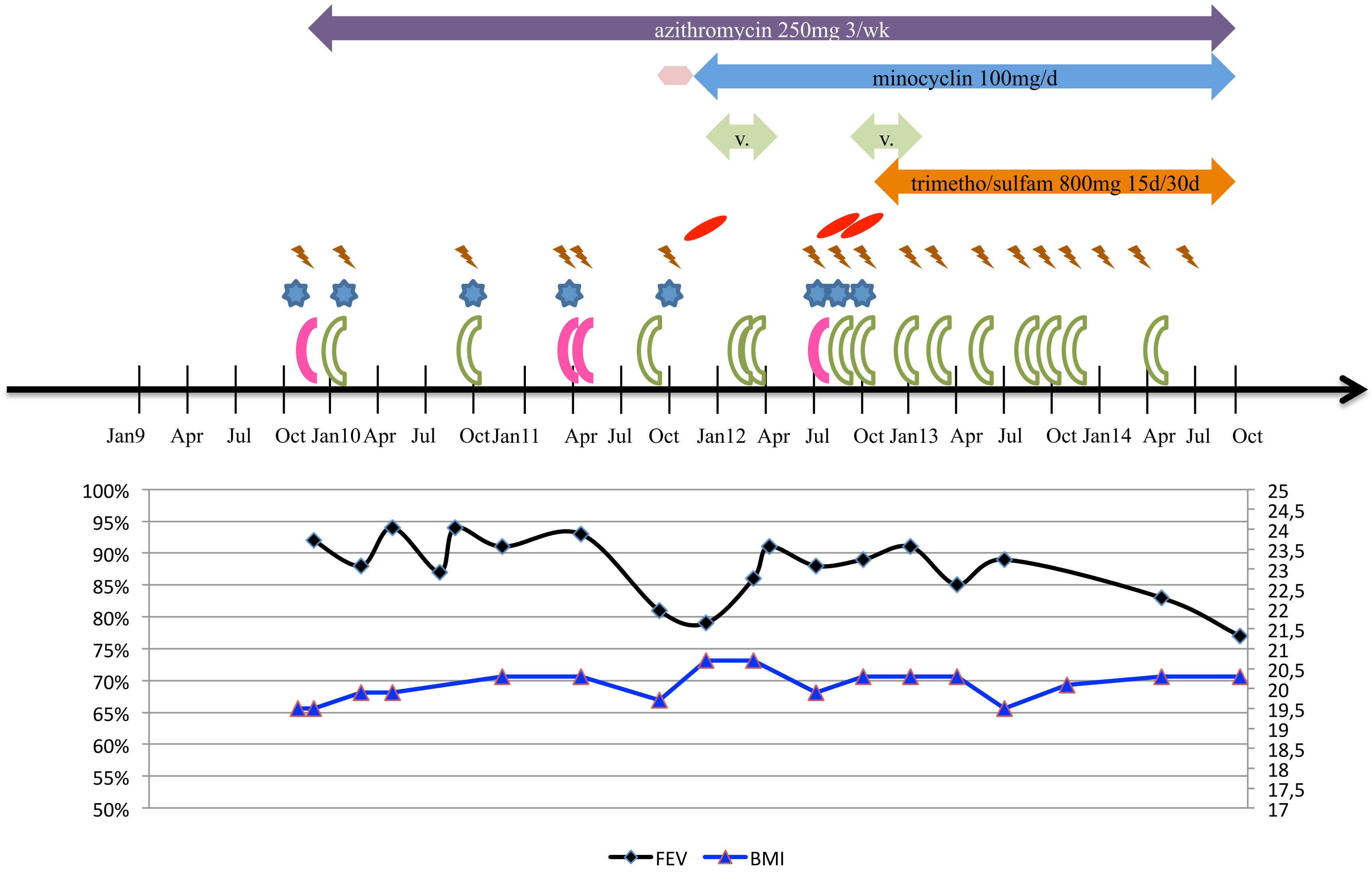

Amoxicillin/clavulanate 15d then oxacillin 15d + ciprofloxacin 1mo

v. Voriconazole 200mg/d
